# Characterization and dynamics of specific T cells against nucleophosmin-1 (NPM1)-mutated peptides in patients with NPM1-mutated acute myeloid leukemia

**DOI:** 10.18632/oncotarget.26617

**Published:** 2019-01-25

**Authors:** Fabio Forghieri, Giovanni Riva, Ivana Lagreca, Patrizia Barozzi, Daniela Vallerini, Monica Morselli, Ambra Paolini, Paola Bresciani, Elisabetta Colaci, Monica Maccaferri, Andrea Gilioli, Vincenzo Nasillo, Andrea Messerotti, Valeria Pioli, Laura Arletti, Davide Giusti, Francesca Bettelli, Melania Celli, Francesca Donatelli, Giorgia Corradini, Sabrina Basso, Antonella Gurrado, Monica Cellini, Tommaso Trenti, Roberto Marasca, Franco Narni, Maria Paola Martelli, Brunangelo Falini, Leonardo Potenza, Mario Luppi, Patrizia Comoli

**Affiliations:** ^1^ Department of Medical and Surgical Sciences, Section of Hematology, University of Modena and Reggio Emilia, Azienda Ospedaliero Universitaria Policlinico, Modena, Italy; ^2^ Department of Laboratory Medicine and Pathology, Unità Sanitaria Locale, Modena, Italy; ^3^ Pediatric Hematology/Oncology Unit, Istituto di Ricovero e Cura a Carattere Scientifico Policlinico San Matteo, Pavia, Italy; ^4^ Cell Factory, Istituto di Ricovero e Cura a Carattere Scientifico Policlinico San Matteo, Pavia, Italy; ^5^ Department of Medical and Surgical Sciences, Section of Pediatric Hemato-Oncology, University of Modena and Reggio Emilia, Azienda Ospedaliero-Universitaria Policlinico, Modena, Italy; ^6^ Institute of Hematology, Centro di Ricerca Emato-Oncologico, University of Perugia, Ospedale S. Maria della Misericordia, S. Andrea delle Fratte, Perugia, Italy

**Keywords:** acute myeloid leukemia, immunity, NPM1 mutation, T cell therapy

## Abstract

Nucleophosmin(NPM1)-mutated protein, a leukemia-specific antigen, represents an ideal target for AML immunotherapy. We investigated the dynamics of NPM1-mutated-specific T cells on PB and BM samples, collected from 31 adult *NPM1*-mutated AML patients throughout the disease course, and stimulated with mixtures of 18 short and long peptides (9-18mers), deriving from the complete C-terminal of the NPM1-mutated protein. Two 9-mer peptides, namely LAVEEVSLR and AVEEVSLRK (13.9–14.9), were identified as the most immunogenic epitopes. IFNγ-producing NPM1-mutated-specific T cells were observed by ELISPOT assay after stimulation with peptides 13.9–14.9 in 43/85 (50.6%) PB and 34/80 (42.5%) BM samples. An inverse correlation between MRD kinetics and anti-leukemic specific T cells was observed. Cytokine Secretion Assays allowed to predominantly and respectively identify Effector Memory and Central Memory T cells among IFNγ–producing and IL2–producing T cells. Moreover, NPM1-mutated-specific CTLs against primary leukemic blasts or PHA-blasts pulsed with different peptide pools could be expanded *ex vivo* from *NPM1*-mutated AML patients or primed in healthy donors. We describe the spontaneous appearance and persistence of *NPM1*-mutated-specific T cells, which may contribute to the maintenance of long-lasting remissions. Future studies are warranted to investigate the potential role of both autologous and allogeneic adoptive immunotherapy in *NPM1*-mutated AML patients.

## INTRODUCTION

Nucleophosmin (*NPM1*) gene mutations, occurring in approximately 30% of adult acute myeloid leukemia (AML) cases, and in 50–60% of AML cases with a normal karyotype, represent one of the most frequent molecular lesions observed in AML [1–3]. Moreover, *NPM1* mutations are specific, being almost exclusively restricted to AML, and usually expressed in the entire leukemic population [1, 4, 5]. As expected for founder genetic lesions, they are also stable throughout the course of the disease, with *NPM1* mutations almost invariably documented in patients experiencing AML relapse [4] Most importantly, *NPM1* mutations result in structural changes of the C-terminus of the NPM1 protein, with subsequent aberrant cytoplasmic delocalization, leading to perturbations in multiple cellular pathways, critical for leukemogenesis [1, 4]. NPM1 cytoplasmic dislocation may also favor protein processing and degradation pathways, presumably leading to more efficient human leukocyte antigen (HLA) presentation [6]. Furthermore, none of the normal human sequences present in databanks match those of the 11 C-terminal residues of the NPM1 mutants, suggesting that this aminoacidic sequence may serve as a leukemia-specific antigen [6]. Based upon the above mentioned biological characteristics, NPM1-mutated protein may therefore be considered an ideal target antigen for AML immunotherapy [7].

Liso *et al*. previously investigated the ability of candidate NPM1-mutated peptides to bind common HLA molecules *in silico* and *in vitro*. They documented that two of the peptides, namely CLAVEEVSL and AIQDLCLAV, both deriving from *NPM1* mutations A and D, bound to HLA-A2 molecules as efficiently as the control peptide derived from the Epstein-Barr virus BMLF1 protein [6]. Furthermore, Greiner *et al*. software-screened the entire aminoacid sequences of the wild-type and mutated (types A, B, C, D) NPM1 protein for HLA-A*0201-binding T-cell epitopes [8]. The ten 9-mer peptides with the highest predictive scores for HLA binding were evaluated in an 8-day culture setting in which peripheral blood (PB) CD8+ T cells were stimulated with antigen presenting cells (APCs) pulsed with individual peptides, and tested for cytokine secretion ability. Two HLA-A2 restricted NPM1-mutated peptides, namely #1 AIQDLCLAV and #3 AIQDLCVAV, were immunogenic, inducing specific T-cell responses in 33% and 44% of *NPM1*-mutated AML patients, respectively. However, only in the case of peptide #3 was the frequency of specific responses in *NPM1*-mutated AML patients statistically higher than in healthy subjects [8]. In addition to these observations, Greiner *et al*. also documented CD8+ T cell cytotoxic activity against either peptide-pulsed T2 cells or primary *NPM1*-mutated leukemic blasts [8]. Intriguingly, in a survival analysis of 25 patients affected with *NPM1*-mutated AML, a better overall survival (OS) was observed in patients with NPM1-mutated-specific T-cell responses, suggesting that immunity against the mutated region of NPM1 may potentially contribute to the favorable clinical outcome of *NPM1*-mutated AML patients [9].

In the current study, we performed extensive immunological examinations to further identify and functionally characterize CD8+ and CD4+ T-cell responses directed towards the NPM1-mutated protein in both PB and bone marrow (BM) samples collected from 31 adult *NPM1*-mutated AML patients at different time points, in order to correlate the dynamics of leukemia-specific immune responses with the clinical course of the disease.

## RESULTS

### Appearance of NPM1-mutated-specific T cells in PB and BM of patients with NPM1-mutated AML and identification of the most immunogenic peptides

**Figure 1:**
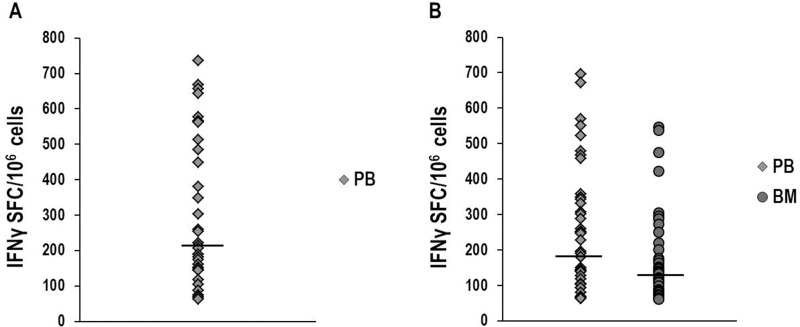
IFNγ-ELISPOT assay to investigate NPM1-mutated-specific T-cell responses Detection of NPM1-mutated-specific T cells producing IFNγ in peripheral blood (PB) and bone marrow (BM) samples, collected at different time-points from *NPM1*-mutated AML patients, after brief *ex vivo* stimulation (20 hours) with NPM1-mutated peptides. The ELISPOT assay, carried out after stimulation with a mixture containing all 18 NPM1-mutated (9–18 mers) peptides, documented NPM1-mutated-specific T cells in 34/52 (65.4%) PB samples (median 214 SFC/10^6^ cells, range 63–736) (Panel **A**). NPM1-mutated-specific T cells were found by ELISPOT assay after stimulation with the combination of 13.9 and 14.9 peptides (Panel **B**), in 43/85 (50.6%) PB samples (median 194 SFC/10^6^ cells, range 62–696) and in 34/80 (42.5%) BM samples (median 133 SFC/10^6^ cells, range 62–546). Median absolute lymphocyte count observed in the analyzed BM samples was 1.9 × 10^9^/L (range 0.2–9.5). Black bars show median values. (*P* value > 0.05, Mann–Whitney *U* Test).

**Figure 2:**
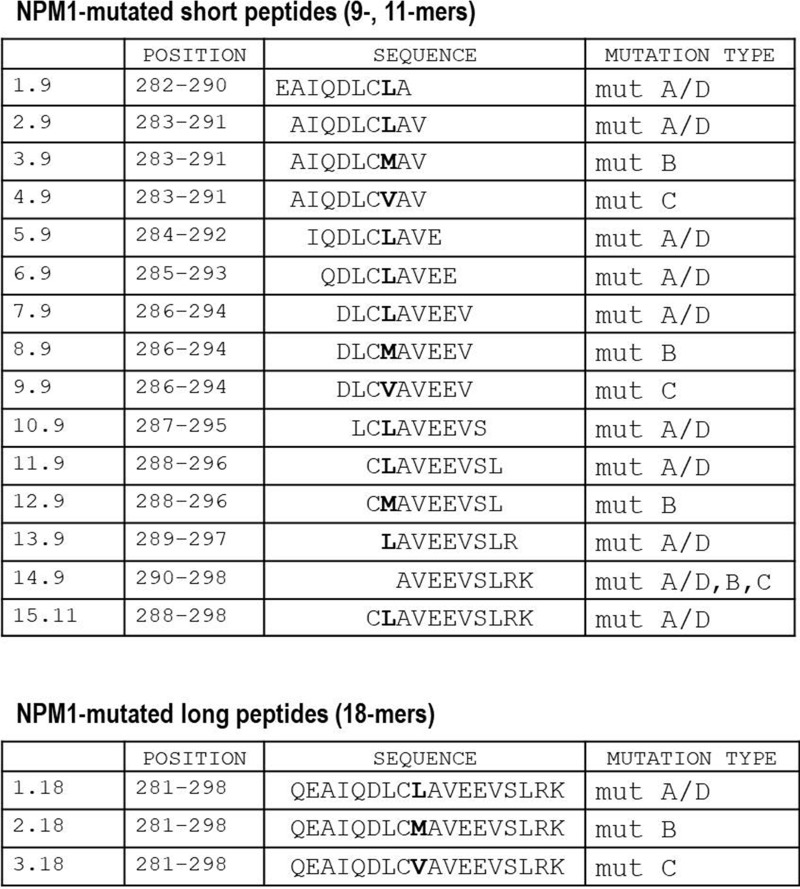
List of NPM1-mutated-derived peptides Position and sequences of 18 peptides deriving from the complete C-terminal of the NPM1-mutated protein, representative of the most common *NPM1* gene mutations, namely A/D, B and C. We designed 15 short (9-, 11-mers) and 3 long (18-mers) peptides. The different aminoacidic residue specific for each mutation type is marked in bold.

We performed IFNγ-ELISPOT assays to investigate the occurrence of NPM1-mutated-specific T cells in a total of 137 PB and 80 BM samples (Table 1), collected at different time points, from 31 adults, affected with *NPM1*-mutated AML (Figure 1). Overall, NPM1-mutated specific immune responses were observed in 26/31 (83.9%) patients. In the initial phase of the study, ELISPOT assays, carried out after antigenic stimulation with a comprehensive mixture containing all of the 18 NPM1-mutated peptides, documented NPM1-mutated-specific T cells producing IFNγ in 34/52 (65.4%) PB samples, obtained from the first 17 patients enrolled in the study (Figure 1A). Subsequent immunological examinations allowed us to identify peptides 13.9 and 14.9 (Figure 2) as the most immunogenic 9-mer peptides within the C-terminal of NPM1-mutated protein. Indeed, NPM1-mutated-specific T cells producing IFNγ were found by ELISPOT assay after brief *ex vivo* stimulation with the combination of 13.9 and 14.9 peptides, in 43/85 (50.6%) PB samples and in 34/80 (42.5%) BM samples, obtained from 26 patients of our series (Figure 1B). No differences in either percentage of positive samples or magnitude of specific immune responses were observed between PB samples stimulated with either peptide mixtures. Moreover, when results from PB and BM samples were compared, no differences were documented (Figure 1B).

**Table 1:** Clinical characteristics of patients with *NPM1*-mutated acute myeloid leukemia and details on samples collected for immunological and molecular monitoring

**Number of patients/Sex**	31 (16 M/15 F)
**Age at AML diagnosis** (years), median (range)	56 (19–75) 10/31 (32.3%) patients aged >60 years
**PB/BM blasts at diagnosis** (%), median (range)	31 (1–90)/60 (20–95)
**WBC count at diagnosis** (× 10^9^/L), median (range)	23.5 (1.1–260)
**Cytogenetics**	25 Normal Karyotype (86.2%) 4 Additional Cytogenetic Abnormalities (13.8%) 2 NA (insufficient karyotype analysis)
***NPM1* mutation type** (*n*° of patients)	A 18/B 3/D 5/others (G, L/Om, ex11, Gm) 4/NA 1
***FLT3* mutational status** (*n*° of patients) (%)	WT 21 (67.8%)/ITD 5 (16.1%)/TKD 5 (16.1%)
Remission induction chemotherapy regimens^*^ (cases) Achievement of CR, cases (%) Consolidation regimens^§^ (cases)	DAE 19/“3+7” 8/MICE 3 27/30 (90%) cytarabine + daunorubicin 22/ICE 3/HDAC 7
**Hematopoietic stem cell transplantation (HSCT)**, cases (%)	autologous HSCT 13 (41.9%) allogeneic HSCT 5 (16.1%) autologous and allogeneic HSCT 3 (9.7%)
**Leukemia relapses** (cases) (%)	16/27 (59.2%)
**Follow-up** (months), median (range)	30 (8–91)
**Alive/dead patients** (cases) (%)	21 (67.7%)/10 (32.3%)
**IFN-γ ELISPOT assay**	
*n*° of analyzed samples (PB/BM)	217 (137/80)
*n*° of timepoints per patient (PB/BM), median (range)	3 (1–8)/3 (1–8)
*n*° of patients with samples collected after induction	16
chemotherapy	80 (52/28)
*n*° of samples collected from 15 patients >12 months after	11
AML diagnosis (PB/BM)	
*n*° of PB samples obtained from healthy subjects	
**Cytokine secretion assay (CSA)**	
*n*° of analyzed samples (obtained from 18 patients)	33
PB/BM (n° of samples)	16 (from 15 patients)/17 (from 12 patients)
*n*° of samples per patient (PB/BM), median (range)	1 (1–2)/1 (1–3)
**MRD monitoring (RQ-PCR mut A, B, D)**	
*n*° of analyzed BM samples (obtained from 18 patients)	102
*n*° of MRD timepoints per patient, median (range)	5 (2–12)

*NPM1*, nucleophosmin 1; AML, acute myeloid leukemia; PB, peripheral blood; BM, bone marrow; WBC, white blood cell; NA, not available; WT, wild type; ITD, internal tandem duplications; TKD, tyrosine kinase domain (D835); IFN-γ ELISPOT, interferon-γ Enzyme Linked Immunospot; MRD, minimal residual disease; RQ-PCR, real-time quantitative polymerase chain reaction.

^*^DAE: daunorubicin 50 mg/m^2^ on days 1, 3, 5, cytarabine 100 mg/m^2^ on days 1–10, etoposide 50 mg/m^2^ on days 1–5; “3+7” regimen: daunorubicin 45 mg/m^2^ on days 1–3, cytarabine 100 mg/m^2^ on days 1–7; MICE: mitoxantrone 7 mg/m^2^ on days 1, 3, 5, cytarabine 100 mg/m^2^ on days 1–7, etoposide 100 mg/m^2^ on days 1–3.

One elderly patient from our series received hypomethylating treatment only with 5-azacitidine 75 mg/m^2^ on days 1–7 every 4 weeks until progression.

^§^cytarabine 500 mg/m^2^ every 12 hours on days 1–6, daunorubicin 50 mg/m^2^ on days 4–6 for 19 adult patients <60 years; daunorubicin 45 mg/m^2^ on days 1–3, cytarabine 100 mg/m^2^ on days 1–7 for 3 elderly patients >60 years; ICE, idarubicin 8 mg/m^2^ on days 1, 3, 5, cytarabine 100 mg/m^2^ on days 1–5, etoposide 100 mg/m^2^ on days 1–3; HDAC, high dose cytarabine 3 gr/m^2^ every 12 hours on days 1, 3, 5.

**Figure 3:**
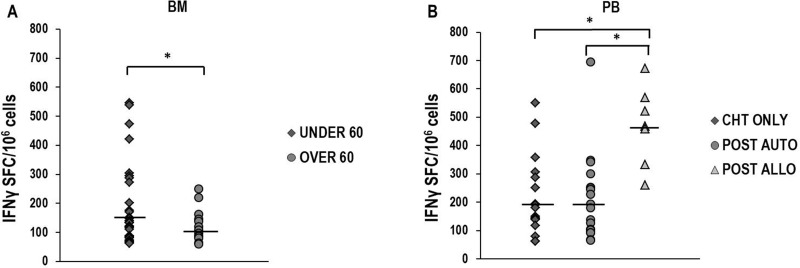
NPM1-mutated-specific immune responses according to patients' age and post-remissional treatments Comparison of IFNγ-producing specific T-cell responses against 13.9 and 14.9 NPM1-mutated-derived peptides in BM samples obtained from 18 younger (median 140 SFC/10^6^ cells, range 62–546) and 8 older than 60 years patients (median 108 SFC/10^6^ cells, range 80–162) (Panel **A**). In panel **B**, the comparisons between IFNγ-producing specific immune responses documented on PB samples from 9 patients who received chemotherapy only (median 180 SFC/10^6^ cells, range 62–550), and from 11 and 6 patients who underwent either autologous (median 180 SFC/10^6^ cells, range 66–696) or allogeneic HSCT (median 468 SFC/10^6^ cells, range 260–672), respectively, are shown. Differences observed were statistically significant (^*^P < 0.05, Mann–Whitney *U* Test). Black bars show median values.

Significantly higher median T-cell responses against 13.9 and 14.9 NPM1-mutated peptides were observed in 52 BM samples from 18 patients younger than 60 years, compared with those documented in 28 BM samples obtained from 8 older patients (*p* = 0.03, Figure 3A). No statistically significant difference was found in younger and older patients when PB specific immune responses were compared (Supplementary Figure 1A), or when immune response to viral antigens, such as CMV, EBV and influenza virus, were evaluated in PB or BM (data not shown). Moreover, we did not document significantly different amounts of specific immune responses when we compared cases according to *FLT3* mutational status (Supplementary Figure 1B, 1C). We also analyzed specific T-cell responses, according to post-remissional therapeutic approaches, comparing samples collected after consolidation with chemotherapy only (9 cases), autologous hematopoietic stem cell transplantation (HSCT) (11 cases) or allogeneic HSCT (6 cases). Interestingly, a significantly higher magnitude of immune response was found in 11 PB samples obtained after allogeneic HSCT, compared with those documented in 37 PB samples collected after chemotherapy only (*p* = 0.01) or 37 PB samples obtained after autologous HSCT (*p* < 0.05). No significant difference was documented between responses found after these two latter consolidation approaches (Figure 3B). Moreover, no statistically significant differences were documented when immune responses evaluated in BM samples were stratified according to post-remission treatments (Supplementary Figure 1D). Intriguingly, after stimulation with the combination of 13.9 and 14.9 peptides, IFNγ-producing NPM1-mutated-specific T cells (median 70 SFC/10^6^ cells, range 68-88) could be revealed by ELISPOT assay in PB samples of 3 out of 11 (27.3%) healthy subjects, tested as controls.

### Correlation between NPM1-mutated-specific immune responses, molecular monitoring and disease course

**Figure 4:**
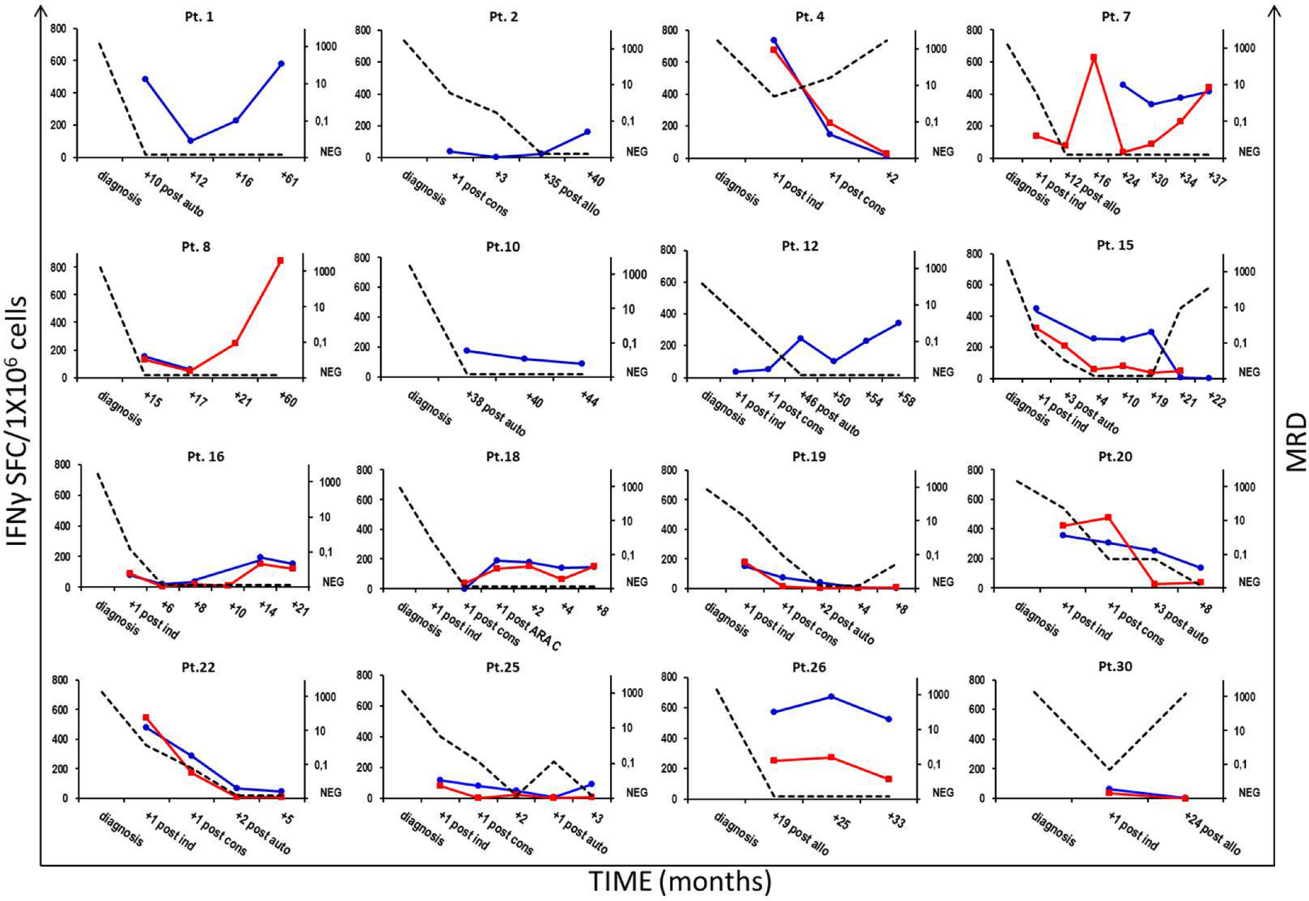
Correlation between NPM1-mutated-specific T-cell responses and AML course Immunological monitoring of NPM1-mutated-specific immune responses in either PB or BM samples from 16 patients with *NPM1*-mutated AML and correlation with the molecular disease course. Frequencies of IFNγ-secreting NPM1-mutated-specific T cells were assessed at different time-points, showing an inverse correlation between NPM1-mutated-reactive T cells and minimal residual disease (MRD) kinetics. Longitudinal data tracking MRD kinetics (right, y-axis; black dotted line) and IFNγ-ELISPOT responses observed in PB and BM samples (left, y-axis, blue and red continuous lines, respectively) are summarized in a single time-course graph for each patient.

IFNγ-producing, specific immune responses were documented early in 10 of the 16 (62.5%) patients, whose samples were available immediately after having achieved morphologic CR with remission induction chemotherapy regimens. Furthermore, high frequencies of IFNγ-producing NPM1-mutated-specific T cells were persistently found in 13 of 15 (86.7%) patients, whose PB (52) or BM (28) samples were collected later than 12 months after AML diagnosis. Molecular MRD monitoring for *NPM1*-mutated transcripts by RQ-PCR was performed on a median of 5 BM samples (range 2–12), obtained at different time points from 18 patients with an *NPM1* gene mutation type A, B, or D. In 12 subjects experiencing long-term morphologic CR, for whom BM samples were available for molecular analysis, MRD was persistently below the cut-off value of 0.1 *NPM1*-mutated/*ABL1*, resulting undetectable in 41 samples (not shown). Paradigmatic clinical courses of 16 patients are reported in Figure 4. Increased and sustained numbers of NPM1-mutated specific T cells were found in patients experiencing persistent molecular CR. Conversely, either a decreased number or absence of IFNγ-producing NPM1-mutated-specific T cells strongly correlated with subsequent molecular or morphologic leukemia relapse, as observed in patients 4, 15, 19, 20 and 30. Similarly, four of the 5 subjects who never exhibited specific immune responses against the NPM1-mutated sequence, namely patients 5, 21, 23 and 28, experienced AML relapse.

### Phenotypic and functional characterization of NPM1-mutated–specific T cells

**Figure 5:**
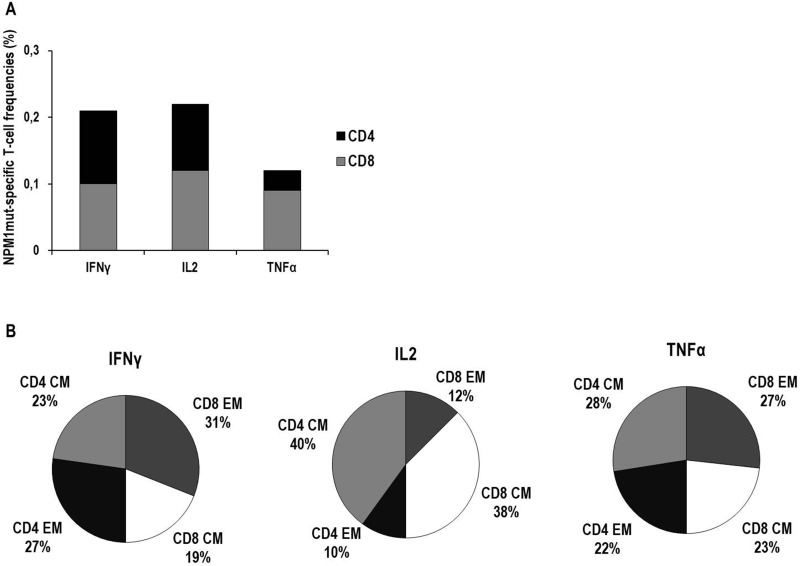
Characterization of cytokine production and memory T-cell profiles of NPM1-mutated specific T cells by cytokine secretion assay (CSA) Frequencies of NPM1-mutated-specific T cells secreting IFN*γ*, IL2 or TNF*α* in BM and PB samples, collected from a total of 18 patients, are expressed as median percentages of CD8+ T cells (gray columns) or CD4+ T cells (black columns) (Panel **A**). In detail, median frequencies (percentage, range) were as follows: IFN*γ*–producing CD8+ T cells 0.1% (0.02–0.84), IFN*γ*–producing CD4+ T cells 0.11% (0.03–0.51), IL2–producing CD8+ T cells 0.12% (0.01–0.62), IL2–producing CD4+ T cells 0.1% (0.01–0.52), TNFα–producing CD8+ T cells 0.09% (0.01–0.91), TNFα–producing CD4+ T cells 0.03% (0.01–0.69). In panel **B**, the memory T-cell profiles of NPM1-mutated-specific T cells, collectively found in PB and BM samples, are shown. For each cytokine-secreting NPM1-mutated-specific T-cell subset, black portions and light gray portions represent the median percentages of CD4+ effector memory (EM) T cells and CD4+ central memory (CM) T cells, respectively, while dark gray portions and white portions represent the median percentages of CD8+ EM T cells and CD8+ CM T cells, respectively.

CSA analysis showed the presence of NPM1-mutated-specific cytokine+ T cells in all of the 33 analyzed samples, as detailed in Table 1 and Figure 5A. The analysis of memory T-cell profiles based on CD62L/CCR7 expression among NPM1-mutated-specific CD8+ and CD4+ T cells is detailed in Figure 5B. Briefly, Central Memory (CM) and Effector Memory (EM) T-cell phenotypes were equally distributed among TNFα–producing T cells, whereas either EM T cells or CM T cells, both CD8+ and CD4+, were predominantly identified among IFN*γ*–producing T cells and IL2–producing T cells, respectively. Of interest, a subset of CD107a+ cytotoxic IFNγ-producing T cells was identified among both CD8+ (median 12%, range 1-67) and CD4+ (median 12%, range 2–49) NPM1-mutated-specific T cells (data not shown).

### Analysis of cytotoxic activity exerted by NPM1-mutated-specific T cells

The 13-day cultures of CTLs, stimulated with NPM1-mutated antigens and tested for lytic activity, included a median 92% (range, 73–98) CD3+ T cells, with 44% (range, 26–56) CD4+ T cells, with either a naïve or CM phenotype (median and range, 48%, 32–69; 22%, 19–45, respectively), and 30% (range, 16–40) CD8+ T cells, with an EMRA phenotype (median and range, 42%, 28–55). CD3−/CD56+ NK cells and CD3+/CD56+ T cells were 6% and 13%, respectively (data not shown).

**Figure 6:**
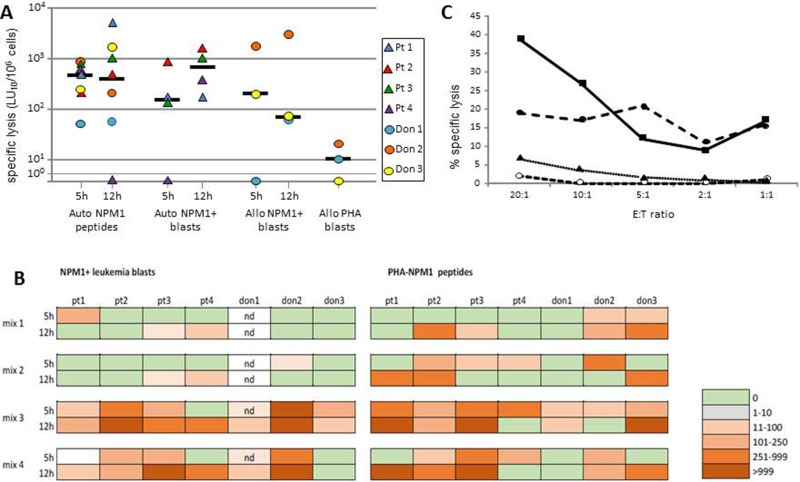
Cytolytic activity exerted by NPM1-specific T cell lines Cytotoxic activity of PBMCs after 13-day culture in the presence of NPM1-mutated–derived peptides against autologous PHA blasts pulsed with NPM1-mutated peptides (Auto NPM1 peptides, 5 h- and 12 h-cytotoxicity assay), autologous *NPM1*-mutated blasts (Auto NPM1 blasts, 5 h- and 12 h-cytotoxicity assay), allogeneic *NPM1*-mutated blasts (Allo NPM1 blasts 5 h- and 12 h-cytotoxicity assay), allogeneic PHA blasts from the donor of the allogeneic *NPM1*-mutated blasts (Allo PHA blasts). The results are represented as the number of lytic units per 10^6^ cells (LU_10_/10^6^) and reported for each subject tested and as median value. LU values referring to lysis of autologous PHA blasts pulsed with NPM1-mutated peptides were calculated after subtraction of background, consisting of cytotoxicity against autologous PHA blasts pulsed with irrelevant peptides (Panel **A**). Cytotoxic responses to *NPM1*-mutated+ autologous (patients) or allogeneic (donors) leukemic blasts or to NPM1-mutated peptide-pulsed PHA blasts in the cultures obtained by stimulation with different NPM1-mutated peptide mixtures (mix 1: peptides 2.9 and 4.9; mix 2: peptides 13.9 and 14.9; mix 3; peptides 13.9, 14.9, and 15.11; mix 4: all 18 peptides) in the subjects tested (Panel **B**). Boxes indicate 5 h- and 12 h-cytotoxicity as LU_10_/10^6^, according to color intensity from green (0 LU_10_/10^6^) to bright orange (>999 LU_10_/10^6^), as reported in the figure. Nd: not done. Cytotoxicity profile of cultured PBMCs obtained from donor 2 (Panel **C**). The figure reports the percentage of specific lysis against autologous PHA blasts pulsed with NPM1-mutated peptides (dashed line, solid circles) or with control peptides (dashed line, open circles), allogeneic *NPM1*-mutated blasts (solid line, squares), and allogeneic PHA blasts from the same donor of the *NPM1*-mutated+ blasts (dotted line, triangles). The mean percent lysis of duplicate wells for 5 different effector-to-target (E:T) ratios is shown.

T cells had a NPM1-mutated–specific lytic activity greater than 100 LU_10_/10^6^ in 6 of the 7 subjects tested (median lysis against NPM1-mutated-derived peptide-pulsed targets: 833 LU_10_/10^6^) (Figure 6A). Control cultures, stimulated with a mixture of 15-mer peptides deriving from viral proteins, did not exhibit lysis greater than 50 LU_10_/10^6^ against target cells pulsed with NPM1-mutated peptides (data not shown). Specific cytotoxicity was mediated by both CD8+ and CD4+ T cells in 6 of 7 subjects, as lysis was observed both with the 5-hr and 12-hr assay, while in one patient NPM1-mutated-specific lysis was exerted exclusively by CD8+ T cells (Figure 6A). Among the different NPM1-mutated-peptide mixtures employed, the combination of 13.9, 14.9, and 15.11 peptides (mix 3) was able to elicit specific responses in all subjects tested, and results were comparable to those observed by using a mix containing all of the peptides (mix 4). The combinations of peptides 2.9 and 4.9 (mix 1) and 13.9 and 14.9 (mix 2) elicited responses in 4 and 6 subjects of the 7 tested, respectively, with responses often below 100 LU_10_/10^6^ or mediated by a single subpopulation of either CD8+ or CD4+ T cells (Figure 6B). We then proceeded to assess whether NPM1-mutated-specific cytotoxic T cells had the ability to recognize and kill leukemia blasts. In 3 of the 4 patients for whom autologous AML blasts were available, we could demonstrate a strong leukemia-directed lytic activity (median lysis, 678 LU_10_/10^6^; Figure 6A, 6C). In 3 healthy donors, we evaluated the response against HLA-partially matched NPM1-mutated-positive AML blasts. A median lysis of 200 LU_10_/10^6^ (range 66–3000) was observed, which was not likely ascribable to alloreactivity, as PHA blasts from the same subjects from whom the tested allogeneic blasts were isolated were not killed (Figure 6A).

## DISCUSSION

In this study, we investigated CD8+ and CD4+ T-cell responses directed towards NPM1-mutated peptides in *NPM1*-mutated AML patients. We observed the spontaneous emergence of T cells secreting IFN*γ* in response to a 18-peptide pool spanning the complete NPM1-mutated protein C-terminal, and identified two peptides, 13.9 and 14.9, as the most immunogenic in our experimental setting. The 14.9 peptide, known to have *in silico* binding affinity at least for HLA A*02:01, A*03:01, A*11:01 and A*68:01, could have a significant advantage based on its extreme C-terminal aminoacidic sequence, that is shared by the most frequently observed *NPM1* mutations, namely A/D, B and C. This could potentially facilitate documentation of specific immune responses, regardless of *NPM1* mutation type [6, 8, 10]. Our ELISPOT assay, performed after brief *ex vivo* antigenic stimulation, differed from the 8-day culture ELISPOT analysis utilized by Greiner *et al*., and this difference may account for different aminoacid sequences being identified as the most immunogenic [8]. In our study, however, the relevance of the identified peptides was confirmed in cytotoxicity experiments, performed after 13-day *in vitro* culture.

In order to expand on the observations by Greiner *et al*. [8, 9], who investigated the presence of NPM1-mutated-specific T cells in a single PB sample per patient, we tested both PB and BM samples, obtained at different timepoints, with the aim of studying the dynamics of specific immune responses throughout the disease course. While the findings did not significantly differ between PB and BM samples, robust NPM1-mutated specific T-cell responses were identified early after reaching CR by remission induction treatments including anthracycline, a prototype of immunogenic chemotherapy [11, 12]. Sustained specific immune responses were commonly observed in patients with long-term CR, whereas either decreased number or absence of NPM1-mutated-specific T cells strongly correlated with leukemia relapse, suggesting an inverse correlation between the kinetics of MRD and anti-leukemic specific T cells, as observed in BCR-ABL-positive acute lymphoblastic leukemia patients [13–15]. The NPM1-mutated-specific immune responses, in some cases observed more than 5 years after the completion of anti-leukemic treatments, may have a central role in the favorable outcome registered in NPM1^mut^/FLT3-ITD^neg^ **v**ersus NPM1 non-mutated AML patients. Greiner *et al*. demonstrated a better overall survival in *NPM1*-mutated AML patients with specific CTL **r**esponses against NPM1-mutated peptides [9]. Indeed, NPM1 mutations are generally present on all leukemic blasts, and leukemic stem cells from NPM1-mutated AML patients carry the mutation [4], thus immune responses directed to NPM1^mut^ may contribute to definitive eradication of MRD. To further reinforce this hypothesis, Kuzelova *et al*. identified a skewed HLA distribution in AML patients, and documented that individuals expressing HLA alleles suitable for presenting NPM1-derived peptides should be less prone to develop *NPM1*-mutated AML.^10^ Indeed, a few HLA class I alleles, mainly B*07, B*18 and B*40, showed a strikingly reduced incidence in the *NPM1*-mutated patient group compared to healthy controls, as well as with *NPM1*-wild type AML patients. Therefore, it has been indirectly hypothesized that specific immune responses to the NPM1 protein may protect a large part of subjects who express appropriate HLA alleles from developing AML, and may help maintain durable responses in the remaining cases, who unfortunately experience AML despite bearing at least one of those depleted alleles. In a larger patient cohort, the same authors subsequently supported the epidemiological hypothesis that anti-NPM1 immune responses could contain AML development and contribute to a better outcome in *NPM1*-mutated AML patients who carry favorable HLA class I types, such as A*02, B*07, B*40 and C*07:01 [16]. Finally, Van Der Lee *et al*. recently demonstrated that various NPM1-mutated-derived peptides are presented on the surface of primary AML cells and that the CLAVEEVSL peptide is a neoantigen which can be efficiently targeted by TCR gene transfer in a co-receptor independent fashion, resulting in TCR-tranduced cell cytolytic capacity against HLA-A*02:01-positive *NPM1*-mutated AML cells [17]. Future studies are warranted to define a protective anti-leukemic cut-off value, and to identify the optimal antigenic burden to mount the highest specific anti-leukemic immune response, as previously demonstrated in chronic myeloid leukemia [18].

NPM1-mutated-specific T-cell responses occurring in PB in the allogeneic HSCT setting were significantly higher than those observed in patients receiving either autologous HSCT or consolidation chemotherapy only. These results are not surprising, since 27.3% of healthy subjects from our series had IFN*γ*-producing NPM1-mutated-specific T-cells, and, in the study by Greiner *et al.*, T-cell responses against NPM1-mutated derived peptides #1 and #3 were documented in 39% and 18% of healthy volunteers, respectively [8]. While it should be acknowledged that these results from *in vitro* assays may potentially reflect the general ability of T cells to be stimulated by a foreign antigen [10], we have further analyzed NPM1-mutated-derived candidate neoepitopes with the comprehensive Immune Epitope Database (https://www.iedb.org/) [19]. Intriguingly, we found that short sequences of four aminoacids from the C-terminal of the NPM1-mutated protein, namely LCLA, CLAV, LAVE, SLRK and VEEV, are homologous with many common bacterial and viral antigens (not shown), suggesting possible cross-reactive immune response mechanisms, as previously documented in melanoma. [20, 21]. This observation may account for the broad immune responses observed in our patients, and may also explain the absence of a significant difference in the levels of these responses in peripheral blood according to age, as the elderly patients in our cohort displayed high levels of antiviral T-cell responses.

In a patient with *NPM1*-mutated AML in molecular relapse after allogeneic HSCT, preemptive donor lymphocyte infusion (DLI) induced polyspecific CD8+ T-cell responses directed also to #1 and #3 NPM1-mutated peptides, which contributed to molecular CR [22]. It was previously described how recipients of an allogeneic HSCT who develop extensive chronic graft versus host disease are able to generate immune responses against wild-type NPM1 [6, 23], while CTL lines deriving from colorectal carcinoma patients may also recognize NPM1, indicating that this protein is immunogenic [24]. Expanding on this observation, Kuzelova *et al*. suggested that the immunogenicity of NPM1-mutated protein is presumably favored by aberrant cytoplasmic localization and also involves several aminoacidic sequences located in the unmutated portion of the protein [10]. Despite the observed NPM1-mutated-specific immune responses in our patients, any evidence of epitope spreading to other antigens, present on leukemic blasts and potentially inducing T-cell responses targeting AML cells, namely WT1, PRAME, RHAMM, proteinase 3 or survivin, cannot be excluded [7, 15, 25, 26].

As previously reported in a different setting [15], by stimulating with dendritic cells pulsed with NPM1-mutated peptides that included the 11-mer CLAVEEVSLRK peptide along with 13.9 and 14.9 epitopes, we were able to expand *ex vivo* leukemia-specific CD8+ and CD4+ CTLs from AML patients, as well as prime leukemia-specific responses in healthy donors. The 11-mer peptide allowed for the stimulation of CD4+ T cells, that have been shown, in addition to providing help to CD8+ CTLs, to mediate potent anti-tumor, HLA class II-mediated, cytotoxic responses *in vivo* [27]. The bulk CD4+ CTLs in our cultures included large numbers of naïve T cells, whose cytolytic activity potential was significantly enhanced after CTLA-4 blockade with checkpoint inhibitors in a mouse model of combined immunotherapy for melanoma [27]. Indeed, leukemic cells, similar to other cancer cells, hijack inhibitory pathways, including activation of immune checkpoints, to evade immune recognition and destruction by CTLs [25, 28]. Therefore, blockade of immune checkpoints has actually emerged as a highly promising approach to increase protective anti-tumor immunity [28–30]. Greiner *et al*. recently investigated the influence of nivolumab and ipilimumab, agents targeting PD-1 and CTLA-4, respectively, on antigen-specific immune responses against AML blasts in functional T-cell assays [31]. Interestingly, the authors documented that the addition of nivolumab to CTL cultures for several days increased both specific T-cell responses against various leukemia-associated antigens, mainly PRAME, RHAMM and WT1, as well as T cell cytotoxic effects against primary AML blasts [31–33]. Furthermore, high PD-L1 expression has recently been documented in *NPM1*-mutated AML cells, especially in leukemic progenitor/stem cell compartments, suggesting that *NPM1*-mutated AML patients may potentially be candidates for immune checkpoint PD-1/PD-L1-driven immunotherapy [34].

In conclusion, we observed the spontaneous development of specific anti-leukemic T-cell immunity, directed against highly immunogenic NPM1-mutated peptides in the majority of patients with *NPM1*-mutated AML, which may contribute to the maintenance of long-lasting CR [9, 13, 35]. Careful monitoring of the correlation between *NPM1*-mutated MRD transcripts and specific T-cell responses against NPM1-mutated peptides, easily evaluable in PB samples, could provide relevant prognostic information [36–38]. In addition to the possibility of generating T cells specifically reactive against patients' primary AML blasts [39, 40]. our data also indicated the feasibility of expanding NPM1-mutated-specific CTL lines from either patients with *NPM1*-mutated AML or healthy donors, who are antigen-naïve. In this latter instance, “neoantigen-specific DLI” to elicit graft-versus-leukemia could be an attractive option for *NPM1*-mutated AML patients, experiencing either morphologic or molecular relapse after allogeneic HSCT [15, 41–45]. Even though adoptive or vaccine immunotherapies are unlikely to be highly effective in patients with full-blown leukemia [25, 28], these strategies could potentially be of value in maintaining CR or, alternatively, eradicating persistent MRD, particularly in elderly subjects, not eligible for allogeneic HSCT [12, 15, 21]. However, prospective studies are warranted to further explore the potential clinical role of individualized anti-leukemic adoptive immunotherapeutic approaches in *NPM1*-mutated AML patients.

## MATERIALS AND METHODS

### Patients and samples

We enrolled a cohort of 31 adult patients (median age 56 years, range 19–75), affected with *NPM1*-mutated AML, and 11 healthy volunteers. Clinical and biological characteristics of the patients are reported in Table 1 and Supplementary Tables 1 and 2. All of the enrolled patients, except for one elderly subject (Pt 29) who underwent hypomethylating treatment with 5-azacitidine, received intensive remission induction chemotherapy; morphologic complete remission (CR) was achieved in 27/30 (90%) of cases. At a median follow-up of 30 months (range 8–91), 16 of the 27 (59.2%) patients experienced leukemia relapse, while, collectively, 21 of 31 (67.7%) patients are still alive, in either first or second CR (Table 1 and Supplementary Table 1). Written informed consent was obtained from the patients and healthy subjects according to the Declaration of Helsinki, after obtaining study approval by the local Institutional Review Board (Comitato Etico Provinciale di Modena–Protocol 4745/13). PB and BM samples were longitudinally collected at different timepoints, starting from morphologic CR.

### NPM1–mutated-derived peptides

NPM1-mutated peptides were synthesized by Biosense to a minimum purity of 70% (immunograde purity) and confirmed by mass spectrometry. All 18 peptides (Figure 2) were utilized as leukemia-specific antigen stimulation for all immunological assays performed in this study, either as mixtures or as individual peptides.

### Enzyme-linked immunospot (ELISPOT) assay

The emergence of NPM1-mutated-specific interferon-γ (IFN-γ)-secreting T cells was investigated by ELISPOT assay, as previously reported [8, 13, 14] and detailed in the Supplementary Methods, on 137 peripheral blood mononuclear cell (PBMCs) and 80 bone marrow mononuclear cell (BMMCs) samples obtained from the enrolled patients (Table 1) and 11 PBMC samples collected from healthy subjects. A total of 2.5 × 10^5^ cells/well were stimulated for 20 hours either with different mixed pools of NPM1-mutated peptides (each peptide used at a final concentration of 50 μg/ml) or with each individual peptide. In detail, IFNγ-ELISPOT assay was initially performed on 52 PB samples, obtained from the first 17 patients enrolled in the study, after antigenic stimulation with a comprehensive mixture containing all of the 18 NPM1-mutated (9–18 mers) peptides. Subsequently, in order to identify the more immunogenic peptides, mixtures were progressively split until single peptides were individually used as antigenic stimulation for the IFNγ ELISPOT assay on the same samples stored from 12 of the 17 patients, previously stimulated with the 18-peptide mixture. After having identified peptides 13.9 (LAVEEVSLR) and 14.9 (AVEEVSLRK) (Figure 2) as the most immunogenic peptides, 14 further *NPM1*-mutated AML patients were enrolled in the study. Collectively, 85 PB and 80 BM samples obtained from 26 patients have been tested by IFNγ ELISPOT assay after stimulation with the combination of 13.9 and 14.9 peptides. Unstimulated PBMCs or BMMCs were used as negative controls, whereas anti-CD3 antibody (Mabtech) was added to positive control wells [13]. Results were considered positive if the number of SFCs/10^6^ cells in NPM1-mutated antigen-stimulated wells was 2-fold higher than that in control wells, and reached at least 60 SFCs/10^6^ cells, according to Cancer Immunoguiding Program guidelines [46] Mann–Whitney *U* test or Wilcoxon signed rank test were performed to compare differences between continuous variables, whereas Chi-square analysis and the Fisher's exact test were used for categorical variables. A value of *p* < 0.05 was considered statistically significant.

### Cytokine secretion assay (CSA)

The functional and phenotypic characterization of NPM1-mutated-specific T cells, stimulated for 3 hours with the 18 NPM1-mutated peptide mixture, was carried out on 33 (16 PB and 17 BM) samples obtained from 18 patients (Table 1) with the cytokine secretion assay (CSA Detection Kit, Miltenyi Biotec, Italy) including the following cytokines: IFNγ, interleukin-2 (IL2) and tumor necrosis factor-α (TNFα), as previously described [13, 14]. The memory phenotype of the cytokine-producing cells was assessed after sample counterstaining with mouse anti-human monoclonal antibody conjugates, as detailed in Supplementary methods. In addition, the cytotoxic phenotype of either CD8+ or CD4+ IFNγ-secreting T cells was assessed by using a monoclonal antibody against the degranulation marker CD107a. Results were expressed as CD8+ or CD4+ T cell percentages [13, 14].

### Minimal residual disease (MRD) monitoring for NPM1-mutated transcripts by reverse-transcriptase quantitative polymerase chain reaction (RQ-PCR)

The levels of *NPM1*-mutated transcripts were analyzed on 102 BM samples collected, at different timepoints, from 18 patients (Table 1 and Supplementary Table 1) [36, 47–49]. The transcriptional expression of *NPM1* gene carrying type A, B, or D mutations was evaluated using Ipsogen *NPM1* mutA, mutB&D MutaQuant diagnostic kits (QIAGEN). RQ-PCR positivity was defined using a threshold cut-off value of 0.1 *NPM1-*mutated*/ABL1* [50].

### Assessment of NPM1-mutated-specific cytotoxic T cells

The presence of specific cytotoxic T-lymphocyte (CTL) subsets was evaluated by co-culturing either PBMCs or BMMCs from 3 healthy donors or patients' (namely patients 2, 7, 15, 16), obtained from single samples resulting positive by the ELISPOT assay, with NPM1-mutated peptide-pulsed autologous dendritic cells, at a responder/stimulator ratio of 20:1. On day 13, responder cells were tested against a panel of targets, including autologous mock-pulsed or NPM1-mutated peptide-pulsed PHA T-cell blasts (PHA blasts), and autologous or allogeneic NPM1-mutated-positive AML blasts. PHA blasts were obtained as previously reported.13

Target cells were incubated overnight with ^51^Cr. For the cytotoxicty assay, effector cells were incubated with target cells at effector/target (E:T) ratios from 10:1 to 0.01:1. Results are reported as percent specific lysis at different E:T ratios, or as lytic units (LUs) [13].

## SUPPLEMENTARY MATERIALS FIGURE AND TABLES





## Abbreviations

NPM: Nucleophosmin
AML: acute myeloid leukemia
PB: peripheral bloos
BM: bone marrow
MRD: minimal residual disease
IFN: interferon
IL: interleukin
PHA: phytohemagglutinin
BMLF1: BamHI-M leftward reading frame 1
APC: antigen-presenting cell
CD: cluster of differentiation
OS: overall survival
HSCT: hematopoietic stem cell transplantation
CR: complete remission
CSA: cytokine secretion assay
CM: central memort
EM: effector memory
LU: lytic unit
TCR: T cell receptor
DLI: donor lymphocyte infusion
CTL: cytotoxic T lymphocyte
PBMC: peripheral blood mononuclear cells
BMMC: bone marrow mononuclear cells
TNFα: tumor necrosis factor-α
