# Supplementary material for: Characterization and dynamics of specific T cells against nucleophosmin-1 (NPM1)-mutated peptides in patients with NPM1-mutated acute myeloid leukemia
Source: Oncotarget. 2019 Jan 25;10(8):869–82. doi: 10.18632/oncotarget.26617 (PMC6368236; doi:10.18632/oncotarget.26617)
Supplement: Supplementary file 1 [file oncotarget-10-869-s001.pdf]

Characterization and dynamics of specific T cells against nucleophosmin-1 (NPM1)-mutated peptides in patients with NPM1-mutated acute myeloid leukemia

SUPPLEMENTARY MATERIALS

Patients and samples

We have enrolled a cohort of 31 adult patients (median age 56 years, range 19–75), affected with *NPM1*-mutated AML, and 11 healthy volunteers. All the enrolled patients, except for one elderly subject (Pt 29) who underwent hypomethylating treatment with 5-azacitidine, received intensive remission induction chemotherapy.

Either bone marrow mononuclear cells (BMMCs) or peripheral blood mononuclear cells (PBMCs) were isolated by Ficoll-Hypaque density gradient centrifugation (Axis-Shield PoC AS, Oslo, Norway), washed twice in RPMI1640 culture medium, and used either fresh or cryopreserved for immunological analyses.

NPM1–mutated-derived peptides

NPM1-mutated peptides have been synthesized by Biosense to a minimum purity of 70% (immunograde purity) and confirmed by mass spectrometry. All the 18 peptides tested were utilized as leukemia-specific antigen stimulation for all the immunological assays performed in this study, either as mixtures or as individual peptides.

Enzyme-linked immunospot (ELISPOT) assay

Cells were counted by the use of automated cell counters (AcT8, Beckman Coulter), resuspended at the appropriate cell density in RPMI1640/fetal calf serum 10% (Invitrogen) and cultured at 37° C in a humidified 5% CO2 atmosphere in 96-well bottom plates coated with anti-IFNγ monoclonal antibodies (Mabtech, Nacka Strand, Sweden).[10]

A total of 2.5×10^5 cells/well were stimulated for 20 hours either with different mixed pools of NPM1-mutated peptides (each peptide used at a final concentration of 50 μg/ml) or with each peptide individually. Unstimulated BMMCs or PBMCs were used as negative controls, whereas anti-CD3 antibody (Mabtech) was added to positive control wells at a final concentration of 5 μg/ml. After incubation, biotinylated secondary antibody (Mabtech, 0.5 μg/ml) was added, and plates were then processed according to standard procedures [10].

The number of spot forming cells (SFCs) per well was quantified using an automated ELISPOT counter (AID-GmbH, Strassberg, Germany). All test conditions were carried out in triplicate and results for individual time-points were calculated as a median value of SFCs obtained after antigenic stimulations compared to control wells and expressed as number of SFCs on 10^6 PBMCs or BMMCs. Results were considered positive if the number of SFCs/10^6 cells in NPM1-mutated antigen-stimulated wells was 2-fold higher than that in negative control wells, and there were at least 60 SFCs/10^6 cells, according to Cancer Immunoguiding Program guidelines (www.cimt.eu/cms/diskfiles/download/7/418244ec26c905deaf7b1e9cc38d004f/cip_guidelines.pdf) Mann-Whitney U test or Wilcoxon signed rank test were performed to compare differences between continuous variables, whereas Chi-square analysis and Fisher's exact test were performed for categorical variables. A value of $p < 0.05$ was considered statistically significant.

Cytokine secretion assay (CSA)

In summary, 1×10^6 PBMCs or BMMCs were cultured at 37° C and stimulated for 3 hours with the 18 NPM1-mutated peptides comprehensive mixture, as used for Elispot assay. In parallel, for each analysis, unstimulated and PHA-stimulated PBMCs or BMMCs were used as negative and positive controls, respectively. Subsequently, samples were first labelled with the Catch Reagent, and thereafter with the Detection Reagent, according to the manufacturer's instructions, in order to detect cytokine-secreting cells.

The memory phenotype of the cytokine-producing cells was assessed after sample counterstaining with the following mouse anti-human monoclonal antibodies conjugates CD3 APC, CD8 FITC or PerCP, CD4 PerCP or APC, CD62L or CCR7 PE (T-cell homing surface markers), allowing the identification of Effector Memory (EM) T cells (CD3+, CD8+ or CD4+, CD62L−/CCR7−) or Central Memory (CM) T cells (CD3+, CD8+ or CD4+, CD62L+/CCR7+) [10, 11].

Cells were acquired on a FACSCalibur flow cytometer (BD Biosciences, San Jose, CA), and analyzed by the use of CellQuest (BD Biosciences) and Summit software (Dako Colorado, Fort Collins, CO). Either

CD8+ or CD4+ T cells were gated on CD3+ events after passing through a small lymphocyte gate and frequencies of antigen-reactive EM or CM T cells were calculated as median differences compared to unstimulated controls.

Minimal residual disease (MRD) monitoring for NPM1-mutated transcripts by reverse-transcriptase quantitative polymerase chain reaction (RQ-PCR)

One microgram of total RNA, extracted from BMMCs, was reverse transcribed in 20 µl reverse-transcriptase reaction. Real-time quantitative PCR was carried out on cDNA on a LightCycler 480 (ROCHE) using the TaqMan method.

NPM1-mutated transcripts levels were compared to expression of *ABL1* (pre-developed assay reagents), used as housekeeping gene. The absolute quantification of *NPM1*-mutated was obtained using a standard curve designed with the fit point method.

Assessment of NPM1-mutated-specific cytotoxic T cells

PBMCs or BMMCs, obtained from single samples found positive by ELISPOT assay, were cocultured with NPM1-mutated peptide-pulsed autologous dendritic cells, at a responder/stimulator ratio of 20:1, in RPMI medium supplemented with 10% fetal calf serum. Dendritic cells were pulsed with different mixtures of all available peptides at the same final concentration used for the IFNγ–ELISPOT assay. Cultures were restimulated on day 7 with 10^6 NPM1-mutated peptide-pulsed, irradiated (3000 rads) autologous PBMCs in the presence of 20 U/mL IL-2 (Novartis, Basel, Switzerland). On day 13, responder cells were tested against a panel of targets, including autologous mock-pulsed or NPM1 peptide-pulsed PHA T-cell blasts (PHA blasts), and autologous or allogeneic NPM1-mutated AML blasts. PHA blasts were obtained as previously reported [10]. Target cells were incubated overnight with ^{51}Cr. For the cytotoxicty assay, effector cells were incubated with target cells at effector/target (E:T) ratios from 10:1 to 0.01:1. Results are reported as the percentage specific lysis at different E:T ratios, or as lytic units (LUs) [10].

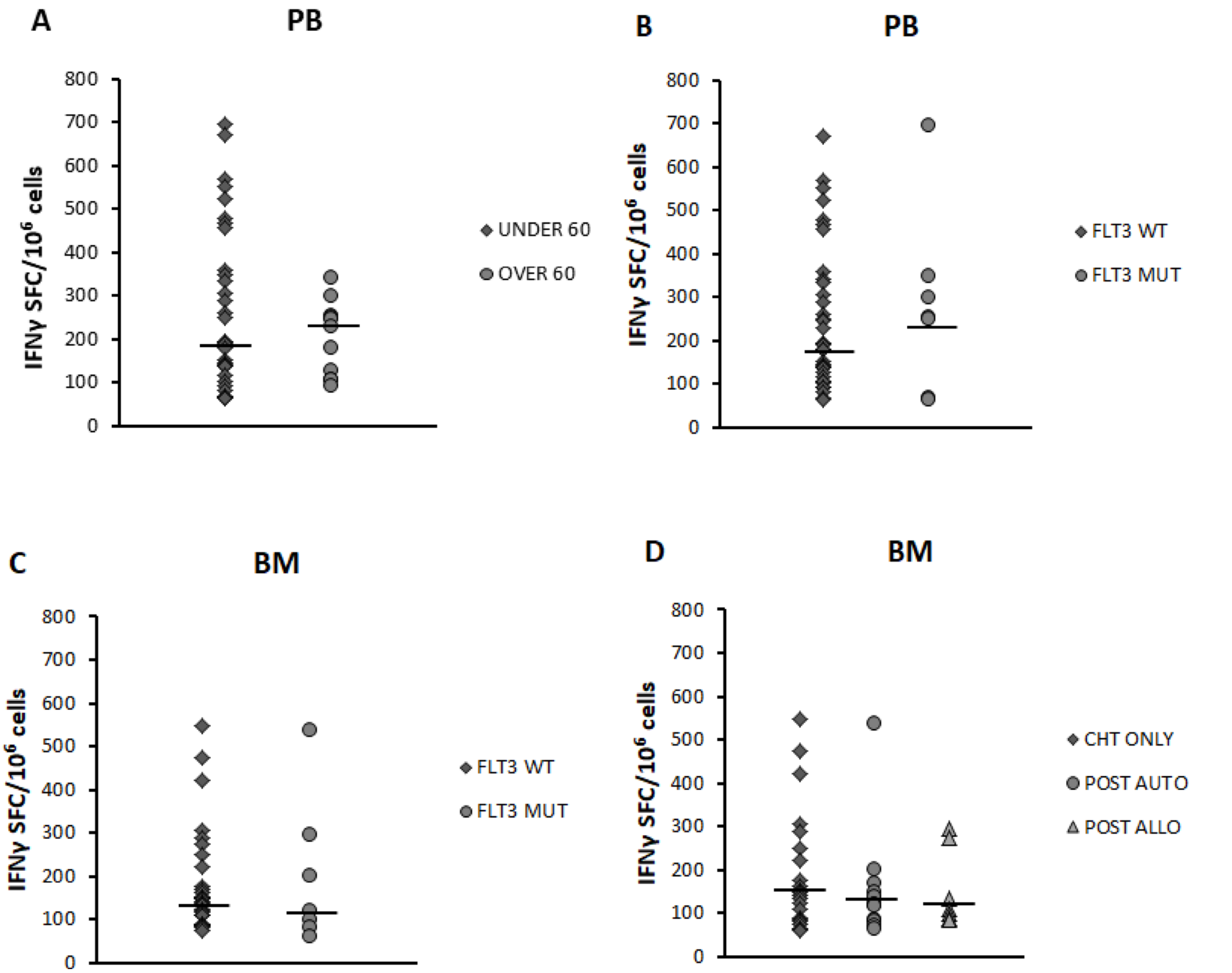

Supplementary Figure 1: Comparison of IFNγ-producing specific T-cell responses against 13.9 and 14.9 NPM1-mutated-derived peptides in peripheral blood (PB) samples obtained from 18 younger (median 194 SFC/10^6 cells, range 62–696) and 8 older than 60 years patients (median 228 SFC/10^6 cells, range 92–342) (Panel A). Panel **B** (PB samples) and C (BM samples) compare specific immune responses in 19 (PB, median 192 SFC/10^6 cells, range 62–672; BM, median 134 SFC/10^6 cells, range 62–546) and 7 (PB, median 252 SFC/10^6 cells, range 62–696; BM, median 120 SFC/10^6 cells, range 80–538) AML patients without or with *FLT3* gene mutations, respectively. In panel **C**, the comparisons between IFNγ-producing specific immune response documented on BM samples from 9 patients having received chemotherapy only (median 148 SFC/10^6 cells, range 62–546), and from 11 and 6 patients who underwent either autologous (median 120 SFC/10^6 cells, range 80–538) or allogeneic HSCT (median 110 SFC/10^6 cells, range 86–272), respectively, are shown. Differences observed were not statistically significant ($P > 0.05$, Mann-Whitney U Test). Black bars show median value.

Supplementary Table 1: Demographics and detailed clinical information on patients with *NPM1*-mutated *AML*. See Supplementary_Table_1

Supplementary Table 2: HLA-typing of patients with specific anti-leukemic T cells, directed against NPM1-mutated peptides

Patient n°	HLA type
Pt 1	HLA-A*26, 29; HLA-B*38, 41; HLA-C*12, 17;<br>HLA-DRB1*04, 11; HLA-DQB1*03, _.
Pt 2	HLA-A*24, 25; HLA-B*18, 65; HLA-C*08, 12;<br>HLA-DRB1*01, 13; HLA-DQB1*05.
Pt 3	HLA-A*02, 32; HLA-B*35, 49; HLA-C*04, 07;<br>HLA-DRB1*14; HLA-DQB1*05.
Pt 4	HLA-A*02, _; HLA-B*44, 47; HLA-C*02, 05;<br>HLA-DRB1*04, 11; HLA-DQB1*03, _.
Pt 5	HLA-A*23, 30; HLA-B*13, 50; HLA-C*06, _;<br>HLA-DRB1*04, 13; HLA-DQB1*03, _.
Pt 6	HLA-A*33:03, 68:01; HLA-B*51:01, 58:01; HLA-C*03:02, 16:02;<br>HLA-DRB1*03:01, 13:01; HLA-DQB1*02:01, 06:03.
Pt 7	HLA-A*24, 26; HLA-B*27, 52; HLA-C*02, 12;<br>HLA-DRB1*13, 15; HLA-DQB1*06, _.
Pt 8	HLA-A*02, 24; HLA-B*14, 18; HLA-C*08, 12;<br>HLA-DRB1*01, 11; HLA-DQB1*03, 05.
Pt 9	HLA-A*02, _; HLA-B*44, 47; HLA-C*02, 05;<br>HLA-DRB1*04, 11; HLA-DQB1*03, _.
Pt 10	HLA-A*01, _; HLA-B*47, 57; HLA-C*06, _;<br>HLA-DRB1*01, 07; HLA-DQB1*05, 03.
Pt 11	HLA-A*30, 32; HLA-B*14, 35; HLA-C*04, 08;<br>HLA-DRB1*13, 14; HLA-DQB1*03.
Pt 12	Not available
Pt 13	HLA-A*32, 68; HLA-B*50, 65; HLA-C*06, 08;<br>HLA-DRB1*13; HLA-DQB1*03, 06.
Pt 14	HLA-A*02:01, 24:02; HLA-B*15:01, 44:02; HLA-C*03:03, 05:03;<br>HLA-DRB1*04:01, 11:04.
Pt 15	HLA-A*23:01, 30:01; HLA-B*18:01, 35:01; HLA-C*04:01, 06:02;<br>HLA-DRB1*08:01, 11:01; HLA-DQB1*03:01, 04:02.
Pt 16	HLA-A*02, 26; HLA-B*27, 57; HLA-C*02, 06;<br>HLA-DRB1*07:01, 16:01; HLA-DQB1*03:03, 05:02.
Pt 17	HLA-A*11, 26; HLA-B*35, _;<br>HLA-DRB1*11, 14; HLA-DQB1*03, 05.
Pt 18	HLA-A*03, 29; HLA-B*44, 51; HLA-C*12, 16;<br>HLA-DRB1*01, 07; HLA-DQB1*02, 05.
Pt 19	HLA-A*01:01, 03:01; HLA-B*18:01, 57:01; HLA-C*06:02, 07:01;<br>HLA-DRB1*11:04, 13:02; HLA-DQB1*03:01, 06:04.
Pt 20	HLA-A*03, 24; HLA-B*35, 44; HLA-C*04, _;<br>HLA-DRB1*01, 11; HLA-DQB1*03, 05.
Pt 21	HLA-A*02:01, 30:02; HLA-B*35:01, 58:01; HLA-C*04:01, 07:01;<br>HLA-DRB1*10:01, 14:01; HLA-DQB1*05:01, 05:03.
Pt 22	HLA-A*11, 25; HLA-B*44, 55; HLA-C*03, 05;<br>HLA-DRB1*14, 16; HLA-DQB1*05, _.
Pt 23	Not available
Pt 24	HLA-A*24:02; HLA-B*39:01, 51:01; HLA-C*01:02, 12:03;<br>HLA-DRB1*04:03, 16:01; HLA-DQB1*03:02, 05:02.
Pt 25	HLA-A*01, 32; HLA-B*35, _; HLA-C*04, 06;<br>HLA-DRB1*11, _; HLA-DQB1*03, _.
Pt 26	HLA-A*23, 24; HLA-B*49, 62; HLA-C*03, 07;<br>HLA-DRB1*11, 13; HLA-DQB1*03, 06.
Pt 27	HLA-A*02, _; HLA-B*44, 47; HLA-C*02, 05;<br>HLA-DRB1*04, 11; HLA-DQB1*03, _.
Pt 28	Not available
Pt 29	Not available
Pt 30	HLA-A*02, 03; HLA-B*39, _; HLA-C*07, 12;<br>HLA-DRB1*08, 13; HLA-DQB1*04, 06.
Pt 31	Not available
