# Supplementary material for: Characterization and dynamics of specific T cells against nucleophosmin-1 (NPM1)-mutated peptides in patients with NPM1-mutated acute myeloid leukemia
Source: Oncotarget. 2019 Jan 25;10(8):869–82. doi: 10.18632/oncotarget.26617 (PMC6368236; doi:10.18632/oncotarget.26617)
Supplement: Supplementary file 2 [file oncotarget-10-869-s002.docx]

**Supplemental table 1. Demographics and detailed clinical information on patients with *NPM1*-mutated AML.**

| **Patient n°** | **Age (ys)/ Sex** | **PB/BM blasts (%)** | **WBC count (x10^9^/L)** | ***NPM1* mutation type** | ***FLT3* mutational status** | **Karyotype** | **Remission induction chemotherapy regimen** | **Achievement of CR** | **Consolidation chemotherapy** | **MRD monitoring/ n° of samples** | **Relapse/ salvage therapy** | **HSCT** | **Follow-up (months)** | **Patient status** |
| --- | --- | --- | --- | --- | --- | --- | --- | --- | --- | --- | --- | --- | --- | --- |
| Pt 1 | 21/M | 17/40 | 17.7 | A | WT | 46,XY | DAE | Yes | Ara-C + daunorubicin | Yes/6 | - | autologous | 67 | alive |
| Pt 2 | 56/M | 70/20 | 52.3 | A | ITD | 46,XY | DAE | Yes | Ara-C + daunorubicin, HDAC | Yes/5 | - | allogeneic in 1^st^ CR | 60 | alive |
| Pt 3 | 66/F | 31/41 | 5.4 | D | WT | 46,XX | MICE | Yes | ICE | - | Yes/ FLAG | - | 12 | dead |
| Pt 4 | 54/F | 53/50 | 87 | A | ITD | 46,XX | 3+7 | Yes | HDAC | Yes/4 | Yes/ FLAG-Ida | - | 8 | dead |
| Pt 5 | 65/F | 54/50 | 45 | A | D835 | 47,XX,+8 | 3+7 | Yes | Ara-C + daunorubicin | Yes/2 | Yes/ FLAG | autologous | 52 | alive |
| Pt 6 | 45/M | 55/50 | 9 | G | D835 | 46,XY | DAE | Yes | Ara-C + daunorubicin, HDAC | - | Yes/ - | - | 15 | dead |
| Pt 7 | 48/F | 9/30 | 25.5 | A | WT | 46,XX | DAE | Yes | Ara-C + daunorubicin | Yes/8 | Yes/5-AZA + DLI | allogeneic in 1^st^ CR | 48 | alive |
| Pt 8 | 58/F | 59/70 | 47.6 | A | WT | 46,XX | DAE | Yes | Ara-C + daunorubicin, HDAC | Yes/5 | - | - | 72 | alive |
| Pt 9 | 34/M | 90/30 | 260 | B | ITD | 46,XY | 3+7 | No | - | Yes/3 | Yes/ FLAG-Ida | allogeneic in CR | 14 | dead |
| Pt 10 | 59/M | 44/35 | 11 | A | WT | 46,XY | DAE | Yes | Ara-C + daunorubicin | Yes/4 | - | autologous | 91 | alive |
| Pt 11 | 67/F | 13/20 | 8.8 | D | WT | 46,XX | MICE | Yes | ICE | - | Yes/ - | autologous | 13 | dead |
| Pt 12 | 65/M | 30/80 | 23 | A | WT | 46,XY, del(13q34) | MICE | Yes | ICE | Yes/2 | - | autologous | 63 | alive |
| Pt 13 | 58/F | 10/20 | 20 | L/Om | WT | 46,XX | DAE | Yes | Ara-C + daunorubicin | - | - | autologous | 60 | alive |
| Pt 14 | 61/F | 1/25 | 1.1 | exon 11 | WT | 46,XX | DAE | Yes | Ara-C + daunorubicin | - | Yes/ FLAG-Ida | autologous | 42 | alive |
| Pt 15 | 66/M | 4/90 | 116 | D | D835 | 46,XY | 3+7 | Yes | Ara-C + daunorubicin | Yes/10 | Yes/ FLAG | autologous | 21 | dead |
| Pt 16 | 43/F | 66/95 | 48 | A | D835 | 46,XX | DAE | Yes | Ara-C + daunorubicin, HDAC | Yes/10 | - | - | 48 | alive |
| Pt 17 | 44/M | 4/70 | 24 | A | ITD | 46,XY, t(6;13;8) | DAE | Yes | Ara-C + daunorubicin, HDAC | - | - | allogeneic/ autologous after graft failure | 78 | alive |
| Pt 18 | 53/F | 3/45 | 4.1 | B | WT | 46,XX | DAE | Yes | Ara-C + daunorubicin, HDAC | Yes/9 | - | - | 30 | alive |
| Pt 19 | 50/F | 84/95 | 133 | D | WT | 46,XX | DAE | Yes | Ara-C + daunorubicin | Yes/5 | Yes/ FLAG-Ida | autologous | 29 | alive |
| Pt 20 | 58/M | 31/80 | 5 | A | WT | 46,XY | DAE | Yes | Ara-C + daunorubicin | Yes/5 | Yes/ FLAG-Ida | autologous | 30 | alive |
| Pt 21 | 42/M | 2/80 | 12.5 | Gm | D835 | 47,XY,+8 | DAE | Yes | Ara-C + daunorubicin | - | Yes/ FLAG-Ida | autologous/ allogeneic in 2^nd^ CR | 19 | dead |
| Pt 22 | 48/M | 90/90 | 91 | A | WT | 46,XY | DAE | Yes | Ara-C + daunorubicin | Yes/4 | - | autologous | 25 | alive |
| Pt 23 | 73/M | 45/50 | 25 | A | WT | 46,XY | 3+7 | Yes | - | - | Yes/ - | - | 12 | dead |
| Pt 24 | 19/M | NA/NA | NA | NA | WT | NA | 3+7 | No | - | - | -/ FLAG-Ida | allogeneic in CR | 43 | alive |
| Pt 25 | 34/F | 62/80 | 103 | A | WT | 46XX | DAE | Yes | Ara-C + daunorubicin | Yes/5 | - | autologous | 24 | alive |
| Pt 26 | 45/F | 10/28 | 8.1 | B | WT | 46,XX | DAE | Yes | Ara-C + daunorubicin | Yes/12 | Yes/ FLAG-Ida | autologous/ allogeneic in 2^nd^ CR | 65 | alive |
| Pt 27 | 60/M | 67/80 | 34.2 | A | WT | NA | DAE | Yes | Ara-C + daunorubicin | - | - | autologous | 56 | alive |
| Pt 28 | 71/F | 7/95 | 2.2 | D | WT | 46,XX | 3+7 | Yes | Ara-C + daunorubicin | - | Yes/ FLAG | - | 25 | alive |
| Pt 29 | 75/F | 4/70 | 2 | A | WT | 46,XX | No (5-AZA) | NE | - | - | - | - | 16 | alive |
| Pt 30 | 44/M | 78/95 | 114 | A | ITD | 46,XY | DAE | Yes | Ara-C + daunorubicin | Yes/3 | Yes/ FLAG-Ida | allogeneic in 2^nd^ CR | 22 | dead |
| Pt 31 | 68/M | 15/70 | 6.8 | A | WT | 46XY | 3+7 | No | - | - | -/ FLAG | - | 8 | dead |

*NPM1*, nucleophosmin 1; AML, acute myeloid leukemia; ys, years; PB, peripheral blood; BM, bone marrow; WBC, white blood cell; NA, not available; NE, not evaluable; WT, wild type; ITD, internal tandem duplications; TKD, tyrosine kinase domain (D835); CR, complete remission; HSCT, hematopoietic stem cella transplantation; MRD, minimal residual disease; DLI, donor lymphocyte infusion.

DAE: daunorubicin 50 mg/m^2^ on days 1,3,5, cytarabine 100 mg/m^2^ on days 1-10, etoposide 50 mg/m^2^ on days 1-5; “3+7” regimen: daunorubicin 45 mg/m^2^ on days 1-3, cytarabine 100 mg/m^2^ on days 1-7.

MICE: mitoxantrone 7 mg/m^2^ on days 1,3,5, cytarabine 100 mg/m^2^ on days 1-7, etoposide 100 mg/m^2^ on days 1-3.

Patient 29 received hypomethylating treatment only with 5-azacitidine 75 mg/m^2^ on days 1-7 every 4 weeks until progression.

Ara-C + daunorubicin: cytarabine 500 mg/m^2^ every 12 hours on days 1-6, daunorubicin 50 mg/m^2^ on days 4-6 for adult patients <60 years; daunorubicin 45 mg/m^2^ on days 1-3, cytarabine 100 mg/m^2^ on days 1-7 for 3 elderly patients >60 years.

ICE, idarubicin 8 mg/m^2^ on days 1,3,5, cytarabine 100 mg/m^2^ on days 1-5, etoposide 100 mg/m^2^ on days 1-3.

HDAC, high dose cytarabine 3 gr/m^2^ every 12 hours on days 1,3,5.

FLAG: cytarabine 2 g/m^2^ on days 1-5, fludarabine 30 mg/m^2^ on days 1-5, filgrastim 5 mcg/kg/day from day -1 to day +5.

FLAG-Ida: cytarabine 2 g/m^2^ on days 1-5, fludarabine 30 mg/m^2^ on 1-5, idarubicin 8 mg/m^2^ on days 1-3, filgrastim 5 mcg/kg/day from day -1 to day +5.
